# Indole alkaloids of *Alstonia scholaris* (L.) R. Br. alleviated nonalcoholic fatty liver disease in mice fed with high-fat diet

**DOI:** 10.1007/s13659-022-00335-2

**Published:** 2022-04-02

**Authors:** Shui-Fen Sun, Hui-Jie Zhong, Yun-Li Zhao, Xiu-Ying Ma, Jin-Bo Luo, Ling Zhu, Yu-Ting Zhang, Wen-Xue Wang, Xiao-Dong Luo, Jia-Wei Geng

**Affiliations:** 1 Department of Infectious Disease and Hepatic Disease, First People’s Hospital of Yunnan Province, Affiliated Hospital of Kunming University of Science and Technology, Kunming, 650032 Yunnan China; 2 School of Medicine, Kunming University of Science and Technology, Kunming, 650500 Yunnan China; 3 Faculty of Life Science and Technology, Kunming University of Science and Technology, Kunming, 650500 Yunnan China; 4 State Key Laboratory of Phytochemistry and Plant Resources in West China, Kunming Institute of Botany, Chinese Academy of Sciences, Kunming, 650201 People’s Republic of China

**Keywords:** Hepatic disease, Hepatic lipogenesis, Fatty acid oxidation

## Abstract

*Alstonia scholaris* (L.) R. Br (Apocynaceae) is a well-documented medicinal plant for treating respiratory diseases, liver diseases and diabetes traditionally. The current study aimed to investigate the effects of TA on non-alcoholic fatty liver disease (NAFLD). A NAFLD model was established using mice fed a high-fat diet (HFD) and administered with TA (7.5, 15 and 30 mg/kg) orally for 6 weeks. The biochemical parameters, expressions of lipid metabolism-related genes or proteins were analyzed. Furthermore, histopathological examinations were evaluated with Hematoxylin–Eosin and MASSON staining. TA treatment significantly decreased the bodyweight of HFD mice. The concentrations of low-density lipoprotein (LDL), triglyceride (TG), aspartate aminotransferase (AST) and alanine aminotransferase (ALT) were also decreased significantly in TA-treated mice group, accompanied by an increase in high-density lipoprotein (HDL). Furthermore, TA alleviated hepatic steatosis injury and lipid droplet accumulation of liver tissues. The liver mRNA levels involved in hepatic lipid synthesis such as sterol regulatory element-binding protein 1C (*SREBP-1C*)*,* regulators of liver X receptor α (*LXRα)*, peroxisome proliferator activated receptor (*PPAR*)γ, acetyl-CoA carboxylase (*ACC1*) and stearyl coenzyme A dehydrogenase-1 (*SCD1*), were markedly decreased, while the expressions involved in the regulation of fatty acid oxidation, *PPARα*, carnitine palmitoyl transterase 1 (*CPT1A*), and acyl coenzyme A oxidase 1 (*ACOX1*) were increased in TA-treated mice. TA might attenuate NAFLD by regulating hepatic lipogenesis and fatty acid oxidation.

## Introduction

Non-alcoholic fatty liver disease (NAFLD) is a chronic hepatic damaging disease and includes fatty liver, non-alcoholic steatohepatitis (NASH), related cirrhosis and hepatocellular carcinoma [1]. NFALD is characterized by serious lipid accumulation, irregular hepatic vein array and hepatic parenchymal cell steatosis that is not the result of excessive alcohol consumption [2]. Obesity and an excessively high-fat diet (HFD) are important factors involved in NAFLD pathogenesis [3]. The prevalence of NAFLD in relation to a HFD is as high as 90% in severely obese individuals [4]. NAFLD is not only associated with metabolic diseases, such as insulin resistance, obesity, diabetes, and hyperlipidemia [5, 6], but also closely related to cardiovascular disease [7], which can seriously harm the health of patients. It is also closely related to hepatocyte injury, which is clinically evaluated via aspartate aminotransferase (AST) and alanine aminotransferase (ALT) levels [8].

Lipid accumulation in the livers of patients with NAFLD usually originates from an imbalance between lipogenesis and lipolysis [9]. Therapeutic drugs for NAFLD sufferers not only remain scarce, but also show some side effects. For example, pioglitazone, a *PPARγ* ligand, induces weight gain, fluid retention, osteopenia and increased fracture risk, especially in older women [10, 11]. Obeticholic acid, a synthetic ligand that activating farnesoid X receptor (FXR), causes both pruritus and moderate increases in low-density lipoprotein (LDL) cholesterol in 25 mg per day doses (NCT02548351) [10, 11]. Thus, exploring novel drugs for NAFLD management by inhibiting the activity of transcription factors related to lipid synthesis has considerable therapeutic potential.

Traditional Chinese medicines for NAFLD has attracted increasing attention in recent decades due to their few adverse effects, proven curative effect and therapeutic mechanisms or benefits [12, 13]. *Alstonia scholaris* is traditionally used to treat respiratory diseases, liver diseases and diabetes in China and Malaysia [14, 15]. The chemical components of different parts of the herb were intensively investigated by our research group [16–37]. More than 100 indole alkaloids were reported, and TA was proved to be the major pharmacological constituents of *A. scholaris* in preventing respiratory diseases by us [38–48]. Both preclinical investigation [49–51] and clinical trials [52, 53] have confirmed that TA was safe for further clinical trials. Besides, the metabolism of TA in rats indicated that scholaricine-type alkaloids could get into circulation more readily than the other types [54]. Hou et al*.,* reported akuammidine, (E)-alstoscholarine, and (Z)-alstoscholarine from TA with nuclear factor-kappa B inhibition [55]. And Shang et al., verified the anti-inflammation activity of TA in mice [56]. Furtherly, Zhao et al., indicated that TA had an inhibitory effect on airway inflammation via regulating the balance of oxidation and anti-oxidation [42]. In addition, A. scholaris decoction is used for treating diabetes, hypertension and malaria [14].

The treatment of NAFLD, a disease related to inflammation and oxidation assumed that TA might have a therapeutic effect on NAFLD, together with its folk use in treating hepatopathy [57–59], then we undertook a pharmacological evaluation of TA on NAFLD in mice induced by high fat diet and explored the potential molecular mechanism.

## Results

### HPLC profile and main constituent contents of TA

**Fig. 1:**
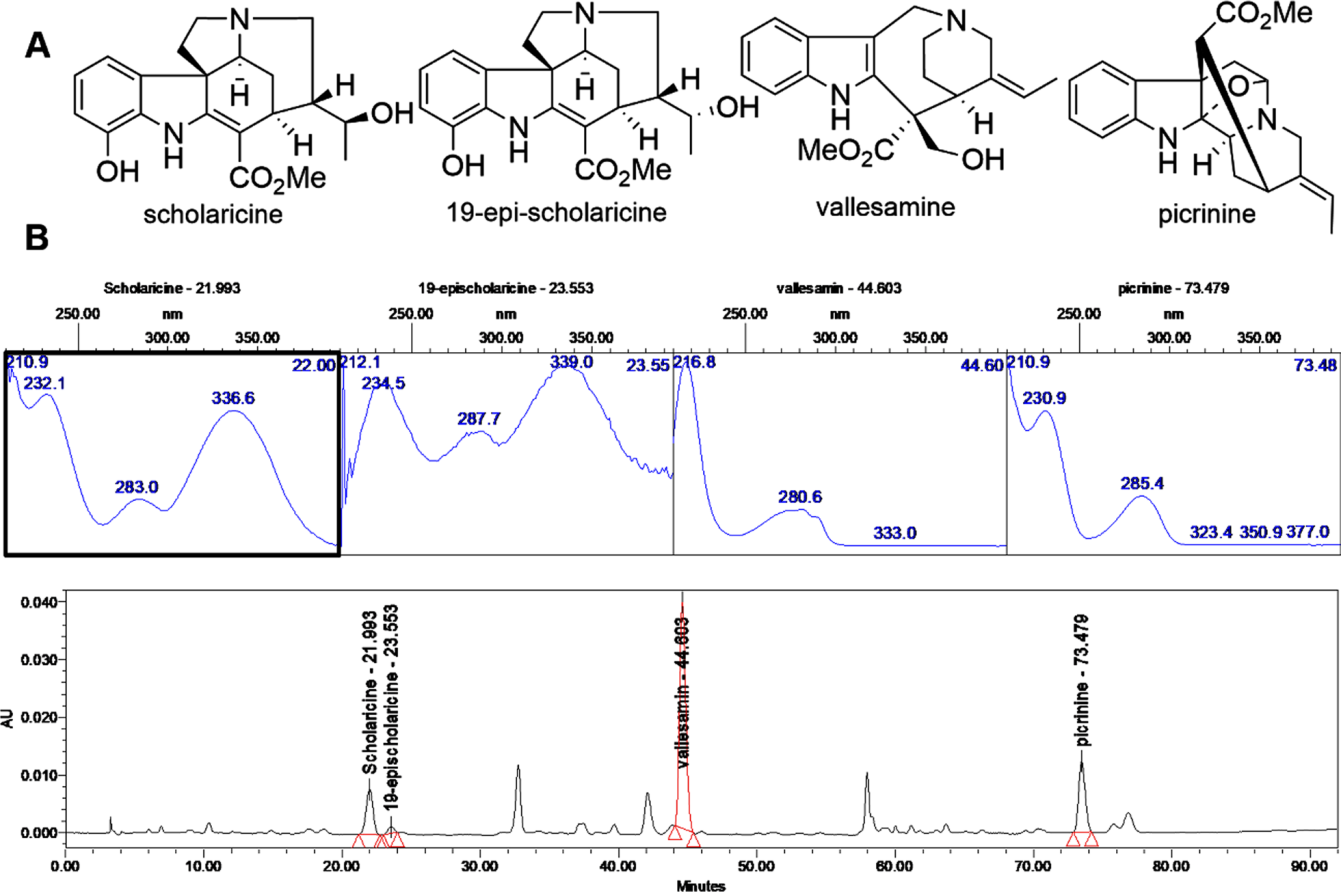
Quantification and identification of TA by HPLC analysis. **A** Structures of four major alkaloids from *A. scholaris*; **B** HPLC chromatographic profile of total alkaloids and UV spectra of four major alkaloids

The main chemical constituents in the TA were separated and identified by high-performance liquid chromatography. Briefly, four chemical components (Fig. 1) were identified as the major medicinal agents in TA, including scholaricine (retention time [RT] = 22.057 min), 19-epischolarine (RT = 23.667 min), vallesamine (RT = 44.915 min), picrinine (RT = 74.29 min), and the contents were 5.26%, 1.13%, 13.91%, 17.39%, respectively.

### TA treatment decreased HFD-induced weight-gaining

After 8 weeks of HFD administration, the HFD-fed mice (31.92 ± 0.62 g) were obviously overweight with the growth rate of 21.46% compared to the normal diet-fed mice (26.28 ± 0.45 g, Table 1). However, HFD-induced weight gain was significantly attenuated in TA (30) dose group (27.88 ± 0.59 g, *P* < 0.01) and the increase amplitude was 12.65%. Silymarin, a routinely used clinical drug for fatty liver and hepatitis, also improved HFD-induced weight-gaining.

**Table 1:** Effects of TA on body weight (BW) in HFD-fed mice at six weeks of treatment

Group	Weight (average ± SEM)
Control	26.28 ± 0.45
HFD	31.92 ± 0.62^▲▲^
HFD + TA (7.5)	29.48 ± 0.53
HFD + TA (15)	31.16 ± 0.28
HFD + TA (30)	27.88 ± 0.59^**/#^
HFD + Silymarin	21.12 ± 1.05

Note: BW was measured in g. Control: mice were fed a normal diet and treated with 0.5% CMC-Na (solvent) via gavage; HFD: mice were fed HFD; HFD + TA (7.5), HFD + TA (15), and HFD + TA (30): mice were fed HFD and treated with TA (7.5, 15, and 30 mg/kg, respectively) via gavage; HFD + Silymarin: mice were fed HFD and treated with silymarin via gavage. ^▲▲^*P* < 0.01 vs. Control; ^**^ *P* < 0.01 vs. HFD group. ^#^ *P* < 0.05 vs. HFD + Silymarin group

### TA treatment improved plasma lipid profiles of HFD-fed mice

As shown in Table 2, HFD-fed mice showed higher TG (from 137.60 ± 24.01 to 222.20 ± 41.27 μg/mL) and LDL (from 96.39 ± 8.18 to 113.70 ± 7.02 μg/mL) levels compared with the control mice. However, TA (30) treatment significantly reduced TG (133.50 ± 21.04 μg/mL) and LDL (84.62 ± 2.82 μg/mL) levels in HFD-fed mice. Furthermore, HDL levels of serum in mice were significantly decreased after 8 weeks HFD intaking (from 90.72 ± 2.08 to 73.89 ± 5.34 μg/mL, *P* < 0.05), which increased partially after TA (30) treatment (83.53 ± 1.87 μg/mL). The treatment of silymarin recovered LDL levels (90.01 ± 13.13 μg/mL) and decreased HDL (75.28 ± 2.61 μg/mL), but had no significant on TG (232.10 ± 21.50 μg/mL, *P* > 0.05). These results suggest that TA treatment can restore normal blood lipid profiles in mice with HFD-intaking. Of note, the reduction of TG in TA (30) group was better than that in the positive control group (*P* < 0.05).

**Table 2:** Effects of TA treatment on blood lipid profiles in HFD-fed mice

Group	TG (average ± SEM, μg/mL)	HDL (average ± SEM, μg/mL)	LDL (average ± SEM, μg/mL)
Control	137.60 ± 24.01	90.72 ± 2.08	96.39 ± 8.18
HFD	222.20 ± 41.27^▲▲^	73.89 ± 5.34^▲^	113.70 ± 7.02^▲^
HFD + TA (7.5)	193.90 ± 18.88	65.72 ± 5.33	98.35 ± 7.55
HFD + TA (15)	207.00 ± 8.88	75.86 ± 7.55	94.47 ± 0.60^*^
HFD + TA (30)	133.50 ± 21.04^**/#^	83.53 ± 1.87	84.62 ± 2.82^*^
HFD + Silymarin	232.10 ± 21.50	75.28 ± 2.61^▲^	90.01 ± 13.13^▲^

TG, HDL, and LDL were measured in μg/mL. Control: mice were fed a normal diet and treated with 0.5%CMC-Na (solvent) via gavage; HFD: mice were fed HFD; HFD + TA (7.5), HFD + TA (15), and HFD + TA (30) mice were fed HFD and treated with TA (7.5, 15, and 30 mg/kg, respectively) via gavage; HFD + Silymarin: mice were fed HFD and treated with silymarin via gavage. ^▲/▲▲^ *P* < 0.05/0.01 vs. Control; ^*/**^ *P* < 0.05/0.01 vs. HFD group. ^#^ *P* < 0.05 vs. HFD + Silymarin group

### TA treatment improves plasma aminotransferase profiles in HFD-fed mice

Following, the plasma levels of AST and ALT were tested to investigate the effects of TA on liver function in mice with HFD. As shown in Table 3, the ALT levels increased significantly from 0.05 ± 0.01 ng/mL of the control group to 0.33 ± 0.04 ng/mL of the HFD group (*P* < 0.01). Similarly, the AST levels also increased significantly from 0.09 ± 0.02 ng/mL to 0.13 ± 0.02 ng/mL (*P* < 0.01). However, these increases were significantly attenuated by the TA treatment (*P* < 0.05/0.01). Interestingly, TA (30) dose-induced improvement in ALT (0.06 ± 0.01 ng/mL) was better than that of silymarin (0.11 ± 0.01 ng/mL, *P* < 0.05), which was an excellent elicitor of liver function repair in clinical practice. Interestingly, TA-induced improvement (0.09 ± 0.03 ng/mL) in AST also exceeded that induced by silymarin (0.10 ± 0.01 ng/mL). Therefore, these results suggest that TA treatment has a significant impact on liver injury induced by HFD.

**Table 3:** Effects of TA treatment on blood aminotransferase levels in HFD-fed mice

Group	ALT (average ± SEM, ng/mL)	AST (average ± SEM, ng/mL)
Control	0.05 ± 0.01	0.09 ± 0.02
HFD	0.33 ± 0.04^▲▲^	0.13 ± 0.02^▲▲^
HFD + TA (7.5)	0.19 ± 0.04^*^	0.09 ± 0.01^*^
HFD + TA (15)	0.13 ± 0.02^**^	0.08 ± 0.02^*^
HFD + TA (30)	0.06 ± 0.01^**/#^	0.09 ± 0.03^*^
HFD + Silymarin	0.11 ± 0.01^**^	0.10 ± 0.01

ALT and AST were measured in ng/mL. Control: mice were fed a normal diet and treated with 0.5%CMC-Na (solvent) via gavage; HFD: mice were fed HFD; HFD + TA (7.5), HFD + TA (15), and HFD + TA (30) mice were fed HFD and treated with TA (7.5, 15, and 30 mg/kg, respectively) via gavage; HFD + Silymarin: mice were fed HFD and treated with silymarin via gavage. ^▲▲^*P* < 0.01 vs. Control; ^*/**^ *P* < 0.05/0.01 vs. HFD group. ^#^ *P* < 0.05 vs. HFD + Silymarin group

### TA treatment alleviates HFD-induced liver injury

**Fig. 2:**
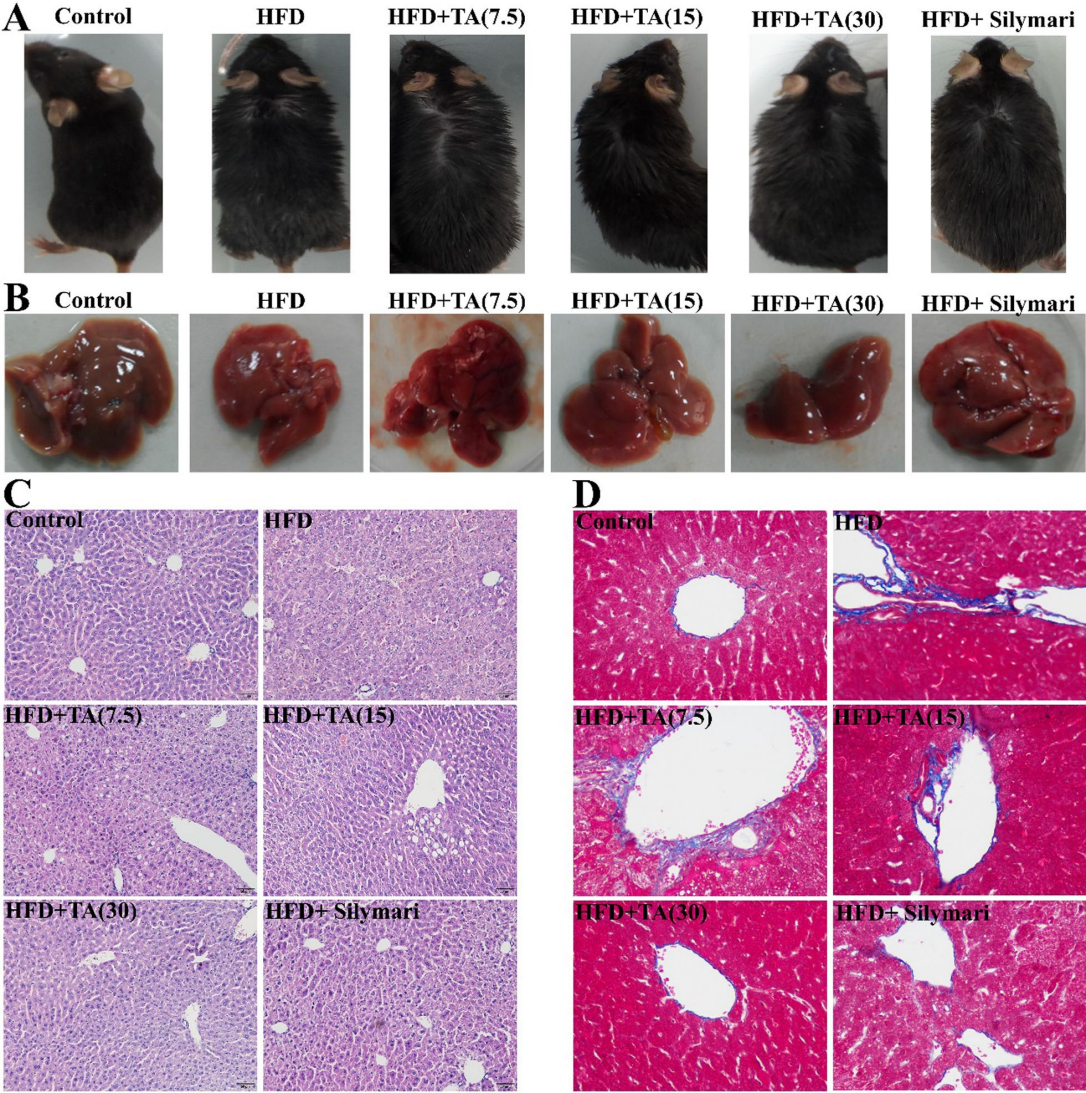
Effect of TA on general observation and histopathologic examination**. A** general observation of mice and livers (**B**), HE (**C**) and Masson (**D**) staining of liver tissues**.** Control: mice were fed anormal diet and treated with 0.5%CMC-Na (solvent) via gavage; HFD: mice were fed HFD; HFD + TA (7.5), HFD + TA (15), and HFD + TA (30) mice were fed a HFD and treated with TA (7.5, 15, and 30 mg/kg, respectively) via gavage; HFD + Silymarin: mice were fed HFD and treated with silymarin via gavage

After HFD administration, mice showed obvious signs of obesity, i.e., 21.46% overweight (Table 1) and greasy hair (Fig. 2A). Of note, TA treatment markedly improved hair condition, especially in the high-dose TA (30) group. The liver tissues of HFD-fed mice showed an obviously rough surface, whereas the liver tissues of normal mice showed a grayish red and glossy surface (Fig. 2B). Interestingly, high-dose TA (30) treatment improved the HFD-induced roughness and redness of the liver surface.

We also performed H&E staining to further confirm the TA-induced histopathological improvement of the livers. As seen in Fig. 2C, normal diet mice showed complete hepatic lobule structures, regular hepatic vein array, and clear cellular structure profiles. In HFD-fed mice, however, the hepatic lobule structure disappeared entirely, hepatic vein array was irregular, and the cell shape and nucleus were blurry. Such liver damage could contribute to the loss of normal lipid metabolic function including lipid synthesis and lipolysis. Furthermore, inflammatory cells penetrated the hepatocyte intervals. Of note, whereas, TA treatment partially repaired the hepatic lobule structure and vein array, especially in the high-dose TA (30) group. Thus, high-dose TA (30) administration not only repaired the hepatic lobule structure and vein array, but also induced lipid droplets to disappear. These TA-induced improvements were comparable to those of silymarin (Fig. 2C). We also applied Masson staining to liver tissue to assess the effects of TA treatment on hepatic fibrosis. As expected, TA treatment remarkably decreased HFD-induced collagen accumulation (Fig. 2D). These results strongly suggest that TA possesses considerable potential in clinical treatment of NFALD.

### TA treatment alters mRNA levels of hepatic fatty acid metabolism-regulating genes in HFD-fed mice

**Fig. 3:**
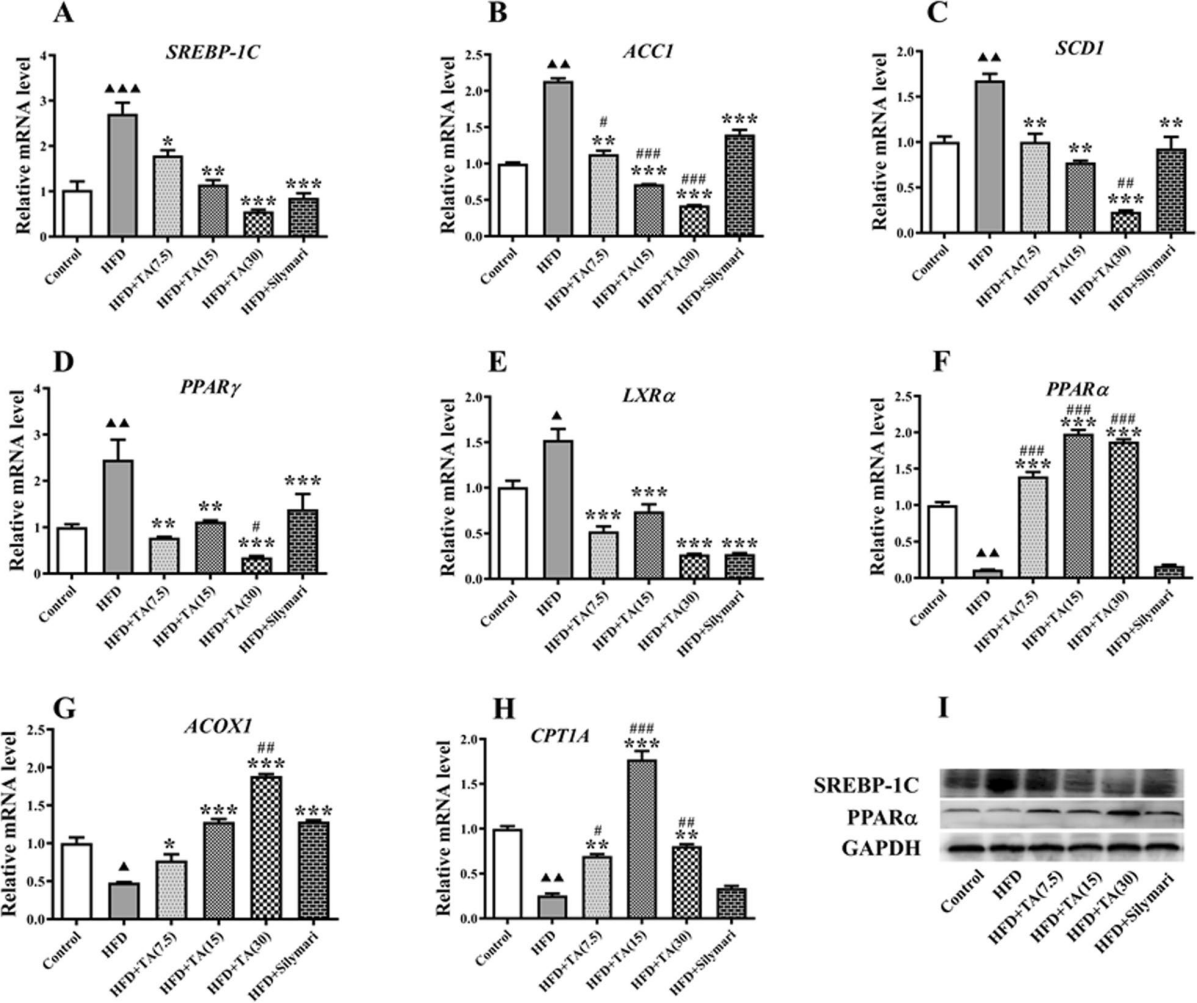
Effect of TA on Lipid metabolism-related gene and protein expressions in liver tissues of HFD-fed mice**.** mRNA levels of *SREBP-1C* (**A**), *ACC1* (**B**), *SCD1* (**C**), *PPARγ* (**D**), *LXRα* (**E**), *PPARα* (**F**), *ACOX1* (**G**), and *CPT1A* (**H**); **I** Protein expressions of *SREBP-1C* and *PPARα*. Control: mice were fed a normal diet and treated with 0.5%CMC-Na (solvent) via gavage; HFD: mice were fed HFD; HFD + TA (7.5), HFD + TA (15), and HFD + TA (30) mice were fed HFD and treated with TA (7.5, 15, and 30 mg/kg, respectively) via gavage; HFD + Silymarin: mice were fed HFD and treated with silymarin via gavage. ^▲/▲▲/▲▲▲^ *P* < 0.05/0.01/0.001 vs. control group; ^*/**/***^ *P* < 0.05/0.01/0.001 vs. HFD group; ^#/##/###^ *P* < 0.05/0.01/0.001 vs. HFD + Silymarin group

As shown in Fig. 3, HFD administration induced high mRNA levels of *SREBP-1C*, *ACC1 SCD1* and *PPARγ*, which are key mediators of lipid synthesis [5, 60, 61]. In addition, *LXRα*, a key mediator of lipid transport, also showed a high mRNA level in HFD-fed mice (Fig. 3E). The expressions of signaling proteins, including *PPARα*, *ACOX1* and *CPT1A*, were decreased in clinical NAFLD samples. We observed similar results in the NAFLD mouse model (Fig. 3F-H), thus supporting the reliability of our model. We previously reported that TA possesses anti-pulmonary fibrosis and pneumonia potential [42, 47, 56]. Therefore, we speculated that TA may have a positive effect on NAFLD and prevent disease progression. As expected, TA treatment significantly attenuated the expression levels of *SREBP-1C*, *ACC1*, and *SCD1* in a dose-dependent manner (Fig. 3A–C). Interestingly, TA-induced attenuations of *ACC1* and *SCD1* were more significant, compared with silymarin (Fig. 3B, C, P < 0.01/0.001). Other contributors to lipid synthesis, namely *PPARγ* and *LXRα*, also showed a significant decrease in mRNA levels, even not in a dose-dependent manner. These results confirm that TA treatment reduces lipid synthesis molecular signaling, especially under high dose conditions (30 mg/kg).

*PPARα*, *ACOX1*, and *CPT1A* maintain lipid metabolism and restrain excessive accumulation of lipids in the liver [62–65]. Here, the mRNA levels of *PPARα* and *ACOX1* decreased markedly following HFD; however, TA treatment restored the mRNA expression levels dose dependently (Fig. 3F, G). Although TA (30) did not show the best performance, all doses of TA induced recovery of *CPT1A* mRNA expression. Of note, the positive control silymarin did not rescue mRNA levels of *PPARα* and *CPT1A* (Fig. 3F and H, P > 0.05). The different performance between silymarin and TA in liver lipid metabolism suggests they may regulate NFALD progression via different molecular mechanisms.

To confirm the TA-induced molecular signaling of lipid metabolism, we applied protein immunoblotting to examine the protein levels of *SREBP-1C* and *PPARα* genes (Fig. 3I). As expected, TA treatment inhibited the expressions of SREBP-1C protein and increased PPARα, respectively, that both were induced by HFD administration (Fig. 3I). Our investigations revealed a mRNA-matched protein levels of *SREBP-1C* and *PPARα* genes, that further confirmed TA management regulates lipid metabolism-molecular signaling during NFALD progression.

## Discussion

A diet high in fat is considered the main cause of NAFLD, and then HFD-fed animal models are often used for studies on NAFLD [66]. In the current research, the bodyweight of mice increased significantly after 8 weeks of HFD administration. The HFD not only increased plasma TG content, but also caused lipid metabolism disorders and significantly increased the accumulation of lipid droplets in the liver. Using this animal model, we investigated whether TA treatment can improve HFD-induced NAFLD and which lipid metabolism signaling was involved in the disease progression.

Lipid metabolism disorders can cause excessive hepatic lipid accumulation. Previous studies have shown that lipid synthesis and uptake-related genes are up-regulated, while genes involved in lipid degradation and secretion are down-regulated in NAFLD [62, 65, 67]. Sterol regulatory element-binding protein 1C (*SREBP-1C*), a key transcription factor of lipogenesis, is activated by upstream regulators of liver X receptor α (*LXRα*), acetyl-CoA carboxylase (*ACC1*) and fatty acid synthase (*FAS*). In NAFLD patients, successive *SREBP-1C* activation originating from *ACC1* and *FAS* can induce hepatic lipid accumulation [61]. Hepatic stearoyl-CoA desaturase 1 (*SCD1*) catalyzes the biosynthesis of monounsaturated fatty acids. Mice lacking *SCD1* are not only significantly less obese than their control counterparts, but also lean and hypermetabolic [5]. In this study, the HFD increased the mRNA levels of *LXRα*, *SREBP-1C*, *ACC1*, and *SCD1* and the SREBP-1C protein in liver tissues. These results are consistent with other studies that describe SREBP activation as essential for hypertriglyceridemia [68]. Our research revealed that TA obtained from the leaves of *A. scholaris* not only inhibited the synthesis of TG, but also decreased the mRNA levels of *LXRα*, *SREBP-1C*, *ACC1*, and *SCD1* and protein level of *SREBP-1C* in a dose-dependent manner.

Furthermore, high levels of *PPAR* gamma (*PPARγ*), a transcriptional modulator of adipocyte development in all types of adipose tissue, promote lipid accumulation [60]. As such, we tested the effects of TA on *PPARγ* expression and found that HFD-induced *PPARγ* activation was also inhibited. Collectively, these results suggest that TA alleviates hepatic fat accumulation in NAFLD by restraining liver lipid synthesis and adipocyte development.

Maintaining liver lipid homeostasis, such as up-regulating fatty acid oxidation (FAO), is essential for reducing liver damage resulting from redundant lipid accumulation [69]. Fatty acid oxidation mainly occurs in the mitochondria with the involvement of peroxisome proliferator-activated receptor α (*PPARα*) [70]. Previous studies have shown that PPARα knockout mice exhibit severe hepatic steatosis accompanied by a decrease in fatty acid uptake and oxidation [71]. The translocation of fatty acid into the mitochondria is dependent on carnitine palmitoyl transferase 1A (*CPT1A*), which is located in the mitochondrial outer membrane [63]. Clinical and animal studies have confirmed that *PPARα*, acyl coenzyme A oxidase 1 (*ACOX1*), and *CPT1A* are significantly down-regulated in NAFLD liver [62, 64, 65]. In the current study, we observed that a HFD altered the expression levels of *PPAR*α and downstream targets *CPT1A* and *ACOX1*. Compared with the control group, the HFD-fed mice showed a significant decrease in the expression levels of lipid synthesis-inhibiting genes, including *PPARα*, *CPT1A*, and *ACOX1*. These results suggest that lipid oxidation is blocked in HFD-fed mice. When HFD group mice were treated with TA, the expression levels of *PPARα*, *CPT1A* and *ACOX1* increased dose dependently. These results indicate that TA may repair lipid metabolism balance in liver tissues of HFD-fed mice by unlocking lipid oxidation.

Chronic lipid accumulation eventually triggers oxidative stress and hepatic injury, which are normally described by AST and ALT values in clinical diagnosis [8]. Our previous studies confirmed that the alkaloid fractions of *A. scholaris* exhibits excellent anti-inflammatory and antioxidant activities, and efficiently inhibits lipid peroxidation [39, 41, 42]. In the present study, the HFD significantly increased the plasma levels of ALT and AST, which were successfully inhibited by TA administration. These results suggest that TA displays the antioxidant and anti-inflammatory effect protecting liver from damage during HFD-induced NFALD progression.

NAFLD increases the risk of cardiovascular disease, and excessive accumulation of TG and LDL can cause atherosclerosis [72]. Furthermore, HDL promotes the induction of anti-atherosclerotic lipoproteins through the reverse transport of cholesterol, thereby effectively preventing cardiovascular diseases [73]. We found that HFD significantly increased the plasma concentrations of TG and LDL and decreased the plasma concentration of HDL, which were, in turn, effectively controlled by TA treatment. Therefore, our research indicates that TA administration may decrease the risk of cardiovascular disease in obese people.

In conclusion, our study demonstrated that TA treatment can successfully ameliorate NAFLD by reducing the expression of the key transcriptional factors *SREBP-1C* as well as the lipogenic enzymes *ACC-1, PPARγ, LXRα* and *SCD-1*. Meanwhile, it upregulated the expression of the lipolytic enzyme *CPT1A*, *PPARα* and *ACOX1*, which are involved in fatty acid oxidation in liver tissues. Therefore, TA have a therapeutic effect on NAFLD through regulating hepatic lipogenesis and fatty acid oxidation.

## Materials and methods

### Preparation of total alkaloids

*A. scholaris* leaves were collected in 2018 in Pu’er city (Yunnan Province, China) and identified by Dr. Xiao-Dong Luo, Kunming Institute of Botany, Chinese Academy of Sciences (Kunming, China). A voucher specimen (Luo20180105) was deposited in the State Key Laboratory of Phytochemistry and Plant Resources in West China, Chinese Academy of Sciences, Kunming, China. The dried and powdered leaves of *A. scholaris* were extracted with 90% EtOH under reflux conditions (3 h X 4), and the solvent was evaporated *in vacuo* to obtain ethanolic extract. Next, the ethanolic extract was dissolved in 0.3% aqueous HCl solution and filtered. The acidic solution was adjusted to pH 9–10 with 10% aqueous ammonia and was extracted with EtOAc to obtain TA fraction, in which picrinine (17.39%), vallesamine (13.91%), scholaricine (5.26%), and 19-epischolaricine (1.13%) were quantified by HPLC with four standard compounds.

### Chemicals

Silymarin was purchased from Madaus AG (Cologne, Germany). Enzyme-linked immunosorbent assay (ELISA) reagents of TG, HDL, LDL, AST, and ALT were purchased from Suzhou Calvin Biotechnology Co., Ltd. (Suzhou, China). RNAiso Plus was purchased from Takara Biotechnology Co., Ltd. (Dalian, China). All primers were synthesized by Sangon Biotech Co., Ltd. (Shanghai, China). GO-Script™ Reverse Transcription Mix and Eastep® qPCR Master Mix were purchased from Promega (Madison, WI, USA). The SDS-PAGE Gel Quick Preparation Kit and Bicinchoninic Acid (BCA) Protein Assay Kit were purchased from the Beyotime Institute of Biotechnology (Jiangsu, China). Antibodies of SREBP-1C and PPARα were purchased from Abcam (Cambridge, MA, USA). The GAPDH antibody and horseradish peroxidase (HRP)-conjugated secondary antibodies were procured from the Proteintech Group Inc. (Chicago, IL, USA) and Thermo Fisher Scientific (Waltham, MA, USA), respectively. High-signal ECL Western Blotting Substrate was purchased from Tanon (Shanghai, China).

### Animals and procedures

Six-week-old C57BL/6 N mice (male) were purchased from Charles River Laboratories (Beijing, China). The high-fat diet (No. D12492) was obtained from Research Diets Inc. (Middlesex County, NJ, USA), and consisted of protein (26.2%), carbohydrate (26.3%), fat (34.9%). All experimental procedures were performed in accordance with the National Institute of Health Guide for the Care and Use of Laboratory Animals. The protocol was approved by the Laboratory Animal Ethics Committee of Kunming University of Science and Technology with approval numbers of 2018GJ512.

Mice were randomly divided into five experimental groups (10 mice/group). The control group was fed a normal chow diet; the HFD group was fed a HFD; and the TA (7.5), TA (15), and TA (30) groups were fed a HFD. After two weeks of HFD, mice in the TA groups were administered (gavage) *A. scholaris*-obtained TA [suspended in 0.5% carboxymethylcellulose sodium (CMC-Na)] at doses of 7.5, 15, and 30 mg/kg body weight (BW), respectively. The positive group mice were fed a HFD, then administered with silymarin by gavage at a dose of 47.8 mg/kg.BW. Both TA and silymarin were administered six days a week for six weeks. After treatment, the mice were fasted for 12 h before sacrifice. Serum was collected from blood obtained by extirpating the eyeballs. Liver tissues were collected and frozen at -80 °C for analysis of gene and protein expression or fixed in 10% formalin for further histopathological analysis.

### Histological analysis

Liver histology was assessed using hematoxylin and eosin (H&E) and Masson stains. Liver tissues were fixed in 10% formalin. The fixed tissues were cut into 5 μm pieces and stained with hematoxylin and eosin (H&E) using standard commercially kits. Masson’s stains in paraffin-embedded sections were furtherly performed using established methodology. Steatohepatitis was defined by the presence of steatosis and inflammation. The severity of steatosis and lobular inflammation were scored using the NASH-Clinical Research Network criteria [3]. Stained samples were observed and photographed using an optical microscope.

### ELISA

Blood was collected via retro-orbital bleeding and centrifuged for 15 min at 1,500 g at 4 °C, then the serum was collected and stored at -80 °C for later analysis. All enzyme-linked immunosorbent assay (ELISA) reagent sets were purchased from Suzhou Calvin Biotechnology Co. Ltd (Suzhou, China), including total triglycerides (TG), high-density lipoprotein (HDL), low-density lipoprotein (LDL), aspartate aminotransferase (AST) and alanine aminotransferase (ALT). Both ALT and AST measurements utilize the Alanine Aminotransferase (ALTP5P) / Aspartate Aminotransferase (ASTP5P) method respectively. The measurement of TG was based on the Fossati three-step enzymatic reaction with a Trinder endpoint. The calculated LDL was determined by subtracting the determined HDL and one-fifth of the triglycerides measured from the total cholesterol.

### Determination of hepatic gene expression based on real-time quantitative polymerase chain reaction (qRT-PCR)

Total RNA was extracted from liver tissues using Trizol reagent according to the manufacturer’s protocols. The concentrations and purities of the RNA samples were then measured. Reverse transcription was performed using the GO-Script™ Reverse Transcription Mix, Oligo (dT). A SYBR Green I Real-Time PCR Kit was used for quantification of *PPARα*, *PPARγ*, *CPT1A*, *ACOX1*, *SREBP-1C*, *ACC1*, *SCD-1, LXRα*, and *GAPDH* mRNA levels using an ABI PRISM 7500 Real-Time System. The amplification reaction conditions were: 95 °C for 5 min, and 35 cycles at 95 °C for 15 s, 60 °C for 30 s, and 72 °C for 1 min. Target gene mRNA levels were compared with *GAPDH* as a reference gene, and the relative quantification of mRNA levels was performed using the 2^−ΔΔCt^ method. Primer sequences used for real-time quantitative PCR are listed in Table 4.

**Table 4:** The primer sequences

Gene	Primer	5′–3′sequence
*LXRα*	Forward	GGGTTGCTTTAGGGATAGG
	Reverse	CATAGCGTGCTCCCTTGAT
*SREBP-1C*	Forward	TTTGCAGACCCTGGTGAGCG
	Reverse	GCAAGACGGCGGATTTATTCA
*ACC1*	Forward	TCTGTATGAGAAAGGCTATG
	Reverse	AAGAGGTTAGGGAAGTCAT
*SCD1*	Forward	GCTCTACACCTGCCTCTTC
	Reverse	CGTGCCTTGTAAGTTCTGTG
*PPARγ*	Forward	GCCCTTTACCACAGTTGA
	Reverse	ACAGACTCGGCACTCAAT
*PPARα*	Forward	CAAGTGCCTGTCTGTCGG
	Reverse	GCGGGTTGTTGCTGGTCT
*ACOX1*	Forward	CTACGCCCAGACGGAGAT
	Reverse	ACGGATAGGGACAACAAA
*CPT1A*	Forward	GGTGTCCAAGTATCTGGCAGTC
	Reverse	TCAGGGTATTTCTCAAAGTCAA
*GAPDH*	Forward	GAGTGTTTCCTCGTCCCG
	Reverse	ATGGCAACAATCTCCACTTT

### Western blot analysis

Protein was extracted from liver tissues using RIPA lysate containing 1% PMSF and quantitated using a BCA Protein Assay Kit. Protein samples were separated by SDS-PAGE and transferred to polyvinylidene difluoride membranes (PVDF). The membranes were first incubated with 5% fat-free milk at room temperature for 3 h, then incubated with antibodies against mouse SREBP-1C (1:5 000), PPARα (1:5 000), and GAPDH (1:5 000) at 4 °C overnight, and finally incubated with corresponding HRP-conjugated secondary antibodies for 1 h at room temperature. Specific bands were visualized by enhanced chemiluminescence (ECL) detection and quantified using ImageJ software. The housekeeping protein GAPDH was analyzed for normalization.

### Statistical analysis

Results are presented as mean ± standard error of the mean (SEM). Statistical analyses were performed using one-way analysis of variance (ANOVA), followed by Tukey’s *post-hoc* test using SPSS 15 software. Differences were considered statistically significant at *P* < 0.05.

## Abbreviations

ACC1: Acetyl-CoA carboxylase
ACOX1: Acyl coenzyme A oxidase 1
ALT: Alanine aminotransferase
ANOVA: One-way analysis of variance
AST: Aspartate aminotransferase
BW: Body weight
CMC-Na: Carboxymethylcellulose sodium
CPT1A: Carnitine palmitoyl transferase 1A
ECL: Enhanced chemiluminescence
FAS: Fatty acid synthase
HDL: High-density lipoprotein
H&E: Hematoxylin and eosin
HFD: High-fat diet
HRP: Horseradish peroxidase
LDL: Low-density lipoprotein
LXRα: Liver X receptor α
NASH: Non-alcoholic steatohepatitis
NAFLD: Non-alcoholic fatty liver disease
PPARα: Peroxisome proliferator activated receptor α
PPARγ: Peroxisome proliferator activated receptor γ
PVDF: Polyvinylidene difluoride membranes
qRT-PCR: Real-time quantitative polymerase chain reaction
SCD1: Stearyl coenzyme A dehydrogenase-1
SREBP-1C: Sterol regulatory element-binding protein 1C
TA: Total alkaloids of *A. scholaris*
TA (7.5): 7.5 Mg total alkaloids per kg body weight
TA (15): 15 Mg total alkaloids per kg body weight
TA (30): 30 Mg total alkaloids per kg body weight
TG: Triglyceride
